# The Association between Cannabis Product Characteristics and Symptom Relief

**DOI:** 10.1038/s41598-019-39462-1

**Published:** 2019-02-25

**Authors:** Sarah S. Stith, Jacob M. Vigil, Franco Brockelman, Keenan Keeling, Branden Hall

**Affiliations:** University of New Mexico, The Department of Psychology, Albuquerque, USA

## Abstract

Federal barriers and logistical challenges have hindered measurement of the real time effects from the types of cannabis products used medically by millions of patients *in vivo*. Between 06/06/2016 and 03/05/2018, 3,341 people completed 19,910 self- administrated cannabis sessions using the mobile device software, ReleafApp to record: type of cannabis product (dried whole natural *Cannabis* flower, concentrate, edible, tincture, topical), combustion method (joint, pipe, vaporization), *Cannabis* subspecies (*C. indica* and *C. sativa*), and major cannabinoid contents (tetrahydrocannabinol, THC; and cannabidiol, CBD), along with real-time ratings of health symptom severity levels, prior-to and immediately following administration, and reported side effects. A fixed effects panel regression approach was used to model the within-user effects of different product characteristics. Patients showed an average symptom improvement of 3.5 (SD = 2.6) on an 11-point scale across the 27 measured symptom categories. Dried flower was the most commonly used product and generally associated with greater symptom relief than other types of products. Across product characteristics, only higher THC levels were independently associated with greater symptom relief and prevalence of positive and negative side effects. In contrast, CBD potency levels were generally not associated with significant symptom changes or experienced side effects.

## Introduction

Medical cannabis markets are currently being flooded with thousands of cannabis strains with unique cannabinoid profiles^1^, novel, uninvestigated cannabis-derived formulates and products with little to no clinical references or formal guidance on how fundamental characteristics of the products themselves may affect pharmacodynamics^2,3^. Federal laws have all but prohibited the use of prospective, pragmatic, naturalistic studies with random treatment assignment for measuring the effects of cannabis consumed *in vivo*. What little clinical research does exist is mostly limited to randomized controlled trials (RCTs) using synthetic cannabinoids or low quality and potency cannabis obtained from the federal government that is unrepresentative of the cannabis products used by millions of people every day^4,5^. Contributing to further confusion are historically contradictory messages coming from the scientific community on the true risks and benefits of cannabis consumption. For example, whereas cannabis was once often and sometimes still is described as component *cause* of schizophrenia^6,7^, several studies now suggest the use of medical cannabis as an effective alternative therapy to antipsychotics and for treating schizophrenia more generally^8–11^. Contradictory effects are often attributed to the distinction between what has been historically interpreted as cannabis’ *harmful*, psychoactive cannabinoid, tetrahydrocannabinol (THC), often described as providing the ‘high’ effects versus the *therapeutic*, non-psychoactive potential (sometimes described as a ‘miracle cure’ in the popular media) of cannabidiol (CBD)^12^. In actuality, few large-scale investigations to date have measured the relative effects of THC and CBD consumption in real-time under naturalistic conditions among people diagnosed with schizophrenia or any other user group.

This is the first study to measure how fundamental characteristics of cannabis products consumed *in vivo* affect immediate symptom relief and experienced side effects. We operationalize our research question using a mobile device software application (app). Although hundreds of cannabis-themed software apps are available for public use^13^, the ReleafApp educational software^14^ is the first app designed specifically to record how the route of administration, combustion method, cannabis subspecies, and major cannabinoid contents are associated with real-time measurements of symptom severity levels, prior to and immediately following administration of cannabis, and the manifestation of myriad possible side effects. Despite recent advocacy for the benefits of CBD over THC, the vast majority of observational studies showing an association between patient-managed cannabis use and improvements in symptoms related to, for example, chronic pain^15^, multiple sclerosis and Parkinson’s disease^16^, post-traumatic stress disorder^17^, and schizophrenia^18^ relied on public or commercially available cannabis that has been hybridized for high THC and low CBD contents, thus suggesting that THC may be an important determinant of user outcomes. Findings from this study are expected to contribute to guidelines for safe and effective cannabis consumption^19,20^, which until now have been limited to anecdotal or retrospective reporting and ungeneralizable experiments.

## Methods

### Study Design

Institutional Review Board approval was obtained from the University of New Mexico for this study and methods were performed in accordance with the approved guidelines. The preexisting anonymized data were obtained with user informed consent through the owner of the ReleafApp, MoreBetter, Ltd., and subject to an investigator confidentiality agreement. The ReleafApp patient education and cannabis treatment management tool was designed to track patient sessions and real-time cannabis use experiences in order to optimize the therapeutic effects of consuming cannabis, while minimizing negative side effects. ReleafApp users voluntarily download the application and enter information on the product they intend to consume, including type of product (whole natural dried flower, concentrate, edible, tincture, and topical); when applicable, combustion method (joint, dry or water pipe, and vape); plant subspecies (*C. indica*, *C. sativa*, or hybrid); and THC and CBD potency levels (percentage of total weight)^21,22^. Testing of the potencies of both cannabinoids is almost universally required under U.S. medical marijuana laws and generally reported on product labels. THC and CBD levels were capped at 35% for flower due to biological limitations on how much THC and CBD a plant can contain. Prior to beginning a session, the patient is required to enter a negative health symptom, selected from 27 possible symptom categories, for which they are attempting to use cannabis therapeutically. (A list of symptom categories and frequencies is available in Supplementary Table S1.)

After entering a symptom, patients are prompted to record a starting symptom level on a visual analogue scale from 0 (no detectable symptom level) to10 (severe). The patient then taps the prompt on the screen to begin the session. From that time until the patient closes the session, they can enter multiple symptom severity levels as frequently as desired. For the current analyses, we include in our sample only patients entering starting symptoms greater than 0 and recording at least one symptom level within 90 minutes of starting the session; we use the last symptom level recorded within that timeframe as the ending symptom level. Our final sample includes 19,910 sessions and 3,341 patients who recorded at least one product characteristic in the ReleafApp between 06/06/2016 and 03/05/2018. Because the entry of product characteristics is voluntary, the sample sizes used in our analyses vary depending on which product characteristics are included. Whole natural dried *Cannabis* flower and concentrates made from the flower are the most common types of products and most likely to contain information on the full spectrum of product characteristics. Panels A through E of Table 1 show descriptive statistics for the product characteristics recorded by the ReleafApp; specifically, product type (Panel A), flower and concentrate combustion method (Panel B), subspecies (Panel C), THC potency (Panel D), and CBD potency (Panel E).

**Table 1:** Descriptive Statistics.

	Mean	Std. Dev.	Minimum	Maximum
**Panel A: Product Type (19,910 sessions, 3,341 users)**				
Concentrate	0.17	0.38	0	1
Edible	0.05	0.21	0	1
Flower	0.74	0.44	0	1
Tincture	0.04	0.20	0	1
Topical	0.00	0.06	0	1
**Panel B: Subspecies (17,197 sessions, 2,996 users)**				
Hybrid	0.48	0.50	0	1
*C. indica*	0.30	0.46	0	1
*C. sativa*	0.22	0.41	0	1
**Panel C: Combustion Method (16,902 sessions, 2,936)**				
Joint	0.13	0.33	0	1
Pipe	0.43	0.49	0	1
Vape	0.45	0.50	0	1
**Panel D: THC (6,958 sessions, 1,260 users)**				
% THC	28.3	22.8	0	100
THC < 10%	0.16	0.36	0	1
THC 10–19%	0.29	0.45	0	1
THC 20–34%	0.36	0.48	0	1
THC 35%+	0.20	0.40	0	1
**Panel E: CBD (5,400 sessions, 1,123 users)**				
% CBD	11.6	16.0	0	100
CBD < 1%	0.25	0.43	0	1
CBD 1–9%	0.33	0.47	0	1
CBD 10–34%	0.34	0.47	0	1
CBD 35%+	0.08	0.28	0	1
**Panel F: Outcome and Control Variables (19,910 sessions, 3,341 users)**				
Symptom Change	−3.5	2.6	−10	9
Starting Symptom Level	6.0	2.2	1	10
Ending Symptom Level	2.4	2.2	0	10
**Panel G: Side Effects (15,617 sessions, 2,757 users)**				
Any Negative Side Effect	0.62	0.48	0.00	1.00
% of Negative Side Effects	0.12	0.14	0.00	0.92
Any Positive Side Effect	0.95	0.23	0.00	1.00
% of Positive Side Effects	0.25	0.18	0.00	1.00
Any Context-Specific Side Effect	0.78	0.41	0.00	1.00
% of Context-Specific Side Effects	0.22	0.20	0.00	1.00

Notes: Nineteen positive, twelve negative, and eleven context-specific side effects were available for selection.

### Study Outcomes

Our main outcome is changes in symptom severity following cannabis consumption (ending symptom level minus starting symptom level) as shown in Table 1, Panel F. During a session, the patient also can report side effects, including 12 negative side effects, 19 positive side effects, and 11 context-specific side effects, crowd-sourced from users, dispensaries, beta testers, and app developers (Supplementary Table S2). Patients can select as many side effects as they like, and at least one side effect was reported in 78% of sessions in our sample. We use as outcome variables whether a patient reported any side effect by category and the percent of the number of side effects available in each of our three respective categories that a patient selected (Table 1, Panel G). The most commonly reported negative side effects are Dry Mouth (26%) and feeling Foggy (23%), the most frequent positive side effects are Relaxed (63%) and Peaceful (54%), and the most common context-specific side effects are feeling High (37%) and Thirsty (27%).

### Statistical Analysis

Our basic statistical model uses a least squares panel regression approach with repeated observations (sessions) at the patient level to analyze how product characteristics affect symptom relief and side effects. We include patient-specific fixed effects to control for time-invariant user characteristics, in order to compare how changing a product characteristic affects symptom relief and side effects for a given user rather than comparing outcomes across users who may differ in many ways, including which products they choose to consume. Because higher starting symptom levels are associated with greater symptom relief (r = 0.60), we control for the starting symptom level in all of our regressions. Standard errors are clustered at the user level to control for heteroskedasticity and arbitrary correlation. Because the number of observations varies substantially by product category, we run regressions separately for each product characteristic category (product type, combustion method, subspecies, and THC and CBD levels) as well as regressions with all product characteristics included.

In order to explore whether cannabis product characteristics differ across symptom categories in their association with momentary symptom relief and side effect profiles, we conduct sub-analyses with samples defined by the three most frequently reported symptom categories: anxiety (16% of the sample), back pain (8%), and depression (10%). Lastly, we explore whether side effect profiles vary with product characteristics. We regress reports of any, and the proportion of side effects selected from each side effect category (negative, positive, context-specific) on our product characteristics using ordinary least squares for consistency across models, although our side effect outcomes are constrained to {0,1} and [0,1]. All predicted outcomes from our regressions fall between zero and one.

## Results

Table 2 shows our results for the effects of product characteristics on patient symptom relief. All regressions control for the starting symptom level, which renders the constant positive. The similar size of the coefficients for the starting symptom level and the constant mean that patients reporting symptom levels of 1 may experience little symptom relief. However, even with a starting symptom level of just 2, users are predicted to experience statistically significant symptom relief. (The average user reports a starting symptom level of 6, SD = 2.2.) The number of sessions, R-squared and number of users are also reported and vary across regressions, with each regression designated by a column.

**Table 2:** Effects of Product Characteristics on Symptom Relief.

Outcome = Symptom Change (Ending - Starting Symptom)					
	(1)	(2)	(3)	(4)	(5)
**Panel A: Product Type, omitted category = flower**					
Concentrate	0.194**				0.080
	(0.076)				(0.212)
Edible	0.340***				
	(0.105)				
Tincture	0.498***				
	(0.117)				
Topical	−0.216				
	(0.355)				
**Panel B: Subspecies, omitted category = hybrid**					
*C. indica*		−0.103**			−0.057
		(0.041)			(0.083)
*C. sativa*		0.096*			0.195
		(0.055)			(0.121)
**Panel C: Combustion Method, omitted category = joint**					
Pipe			−0.061		−0.004
			(0.084)		(0.194)
Vape			0.051		0.051
			(0.092)		(0.210)
**Panel D: THC and CBD, omitted categories = THC < 10% and CBD < 1%**					
THC 10–19%				−0.215*	−0.220*
				(0.117)	(0.134)
THC 20–34%				−0.235**	−0.315***
				(0.112)	(0.121)
THC 35%+				−0.252**	−0.342**
				(0.126)	(0.166)
CBD 1–9%				−0.089	−0.026
				(0.121)	(0.126)
CBD 10–34%				0.038	0.079
				(0.105)	(0.092)
CBD 35%+				−0.222	−0.241
				(0.209)	(0.227)
Starting Symptom Level	−0.704***	−0.721***	−0.726***	−0.709***	−0.717***
	(0.028)	(0.031)	(0.032)	(0.056)	(0.063)
Constant	0.588***	0.691***	0.719***	0.909***	0.850**
	(0.165)	(0.184)	(0.212)	(0.340)	(0.403)
Number of sessions	19,910	17,197	16,898	4,439	3,869
R-squared	0.330	0.340	0.338	0.337	0.346
Number of users	3,341	2,996	2,936	900	787

Notes: Regressions control for individual user fixed effects. Concentrate is relative to Flower, *C. indica* and *C. sativa* are relative to Hybrid, THC categories are relative to THC 0–9%, CBD categories are relative to CBD 0%, and Pipe and Vape are relative to Joint. Standard errors are clustered at the user level (shown in parentheses). ***p < 0.01, **p < 0.05, *p < 0.1.

The first column showing the effects of consuming the different product types on symptom relief relative to the effects from flower suggests that flower provides more symptom relief than any other type of product. The second column comparing *C. indica* and *C. sativa* plant sub-species to hybrid plants suggests that products from pure indicas may increase symptom relief while sativa strains may decrease it. Combustion method does not explain any difference in symptom relief across products within users, as shown in Column [3]. Column [4] of Table 2 shows that whereas higher THC offers greater symptom relief, higher CBD offers no statistically significant benefit. The last column of Table 2 includes all product characteristics. Due to the inclusion of combustion method, only flower and concentrate product types are included. The effect of THC becomes stronger and is the only statistically significant determinant of symptom relief among the product characteristics.

**Figure 1:**
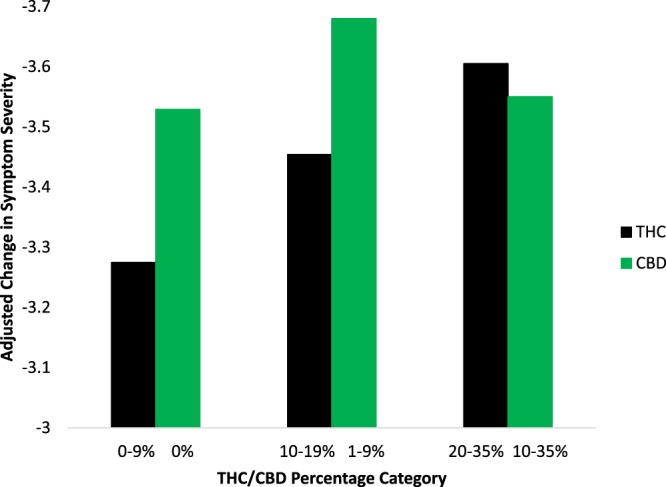
Adjusted Change in Symptom Severity by THC and CBD Percentage Category in Flower.

**Figure 2:**
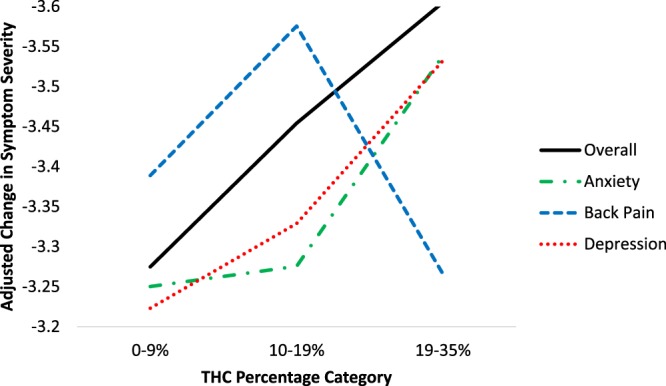
Adjusted Change in Symptom Severity by THC Percentage Category in Flower & Symptom Type.

Figure 1 shows changes in symptom severity by THC and CBD percentage category for dried natural flower, the most popular and homogenous type of cannabis product in the sample, after adjusting for the remainder of the product characteristics. Regression coefficients (controlling for the remaining product characteristics; see Supplementary Table S3) showed that flower containing the middle (10–19%) and highest (20–35%) THC potency levels was associated with greater symptom improvement than flower in the lowest THC potency category (0–9%). As with the omnibus tests in Table 2, variability in CBD levels in flower was not associated with differences in symptom improvement. Figure 2 shows adjusted changes in symptom severity by the THC percentage category of flower run separately for the three most common medical cannabis patient conditions, anxiety, back pain, and depression. Regression coefficients controlling for the remaining product characteristics showed that only users treating depression showed greater symptom improvement from using flower in the middle and highest THC potency categories relative to the least potent flower. In contrast, variability in THC was not associated with statistically significant differences in symptom relief for back pain or anxiety beyond its overall effect on mean symptom improvement levels (see Supplemental Table S3).

In Table 3, we present the relationship between product characteristics and side effect profiles. We restrict our analysis to only concentrates and flower to capture the relationship between different combustion methods and side effects. While these methods do not explain any statistically significant variation in symptom relief, they may be associated with different side effect profiles. We run our full model using any and the percent of each category of side effects selected by the user. Concentrates showed a weaker association with positive side effects, but do not appear to differ from flower in their association with negative or context-specific side effect reporting. Indica-based products are associated with a greater likelihood of reporting negative side effects and some evidence of fewer positive and more context-specific side effects, relative to hybrid- and sativa-based products. Higher THC generally is associated with increased reporting of all three types of side effects. Just as with symptom relief, CBD appears to have little effect on side effect reporting of any kind. There is some evidence that vaping is associated with decreased reporting of negative side effects relative to smoking joints. Lastly, in order to show that a handful of very frequent ReleafApp users are not driving the results, we replicated our main results from Table 2 including only individuals who completed ten sessions or fewer, and separately, including only the first five sessions for all users. THC again is the greatest predictor of symptom relief among the product characteristics. (See Supplemental Table S4 for detailed results).

**Table 3:** Relative Associations of Product Characteristics with Side Effects.

Variables	(1)	(2)	(3)	(4)	(5)	(6)
	Negative	% of Negative	Positive	% of Positive	Context-Specific	% of Context-Specific
Concentrate	−0.024	0.008	−0.097**	−0.090**	−0.052	−0.001
	(0.080)	(0.028)	(0.044)	(0.037)	(0.101)	(0.062)
*C. indica*	0.079**	0.015*	0.011	−0.033**	0.030	0.045***
	(0.035)	(0.009)	(0.017)	(0.016)	(0.026)	(0.010)
*C. sativa*	0.003	−0.002	−0.000	−0.022	−0.041	−0.043**
	(0.045)	(0.008)	(0.012)	(0.016)	(0.033)	(0.019)
Pipe	−0.109	−0.022	−0.046	0.002	−0.041	−0.020
	(0.077)	(0.024)	(0.060)	(0.021)	(0.045)	(0.035)
Vape	−0.168**	−0.037	−0.044	−0.004	−0.050	−0.047
	(0.076)	(0.024)	(0.049)	(0.024)	(0.059)	(0.031)
THC 10–14%	0.113***	0.013	0.017	0.055***	0.178***	0.066***
	(0.043)	(0.010)	(0.018)	(0.015)	(0.050)	(0.020)
THC 15–34%	0.090*	0.016	0.013	0.069***	0.203***	0.085***
	(0.053)	(0.013)	(0.019)	(0.018)	(0.051)	(0.021)
THC 35%+	0.198**	0.050**	0.065*	0.118***	0.182**	0.095*
	(0.091)	(0.025)	(0.036)	(0.033)	(0.072)	(0.052)
CBD 1–9%	0.030	−0.007	0.000	−0.051***	−0.007	−0.009
	(0.042)	(0.014)	(0.015)	(0.018)	(0.038)	(0.024)
CBD 10–34%	0.027	−0.012	0.011	−0.032*	−0.013	0.005
	(0.029)	(0.008)	(0.013)	(0.018)	(0.047)	(0.028)
CBD 35%+	0.042	−0.017	0.016	−0.033	0.093	−0.027
	(0.062)	(0.022)	(0.024)	(0.022)	(0.070)	(0.053)
Starting Symptom Level	0.005	0.003**	0.001	−0.003	0.007	0.003
	(0.007)	(0.001)	(0.002)	(0.002)	(0.005)	(0.002)
Constant	0.566***	0.108***	0.988***	0.288***	0.629***	0.160***
	(0.081)	(0.023)	(0.048)	(0.029)	(0.077)	(0.039)
Observations	3,220	3,220	3,220	3,220	3,220	3,220
R-squared	0.016	0.015	0.009	0.047	0.025	0.052
N Users	665	665	665	665	665	665

Notes: Regressions control for individual user fixed effects. The Concentrate is relative to Flower, *C. indica* and *C. sativa* are relative to Hybrid, THC categories are relative to THC between 0 and 10%, and CBD categories are relative to 0% CBD, and Pipe and Vape are relative to Joint. Standard errors are clustered at the user level (shown in parentheses). ***p < 0.01, **p < 0.05, *p < 0.1.

## Discussion

Given just how common it is for cannabis patients to try different types of products and methods of administration^23,24^, it is surprising how few previous investigations have examined which fundamental characteristics of the products consumed *in vivo* by millions of people daily are associated with real-time patient outcomes and experienced side effects. While RCTs may be the ‘gold standard’ for measuring the pharmacodynamics of synthetic, standardized (usually particular symptom focused) medications, they are poorly suited for understanding the effects of a medication with substantial heterogeneity in product characteristics and consumption methods across the estimated 2.2 million state-legal medical cannabis patients in the United States^25^. Our observational study using mobile app technology was designed to measure these effects in real-time among a large sample of patients using cannabis for treating their medical symptoms under naturalistic conditions. On average, responders experienced significant improvements across the 27 health symptom categories measured. Dried, whole natural flower was associated with greater symptom relief than the use of other types of products (i.e., concentrates, edibles, tinctures, and topicals). However, and despite the fact that different routes of administration deliver variable amounts of cannabinoid contents and have different metabolomics^26–31^, we did not find variation in symptom relief with use of pipes, joints, or vaporization combustion devices. Products made from pure *C. indica* strains were more effective than products made from *C. sativa*, matching patient-reported preferences for the former for treating conditions such as pain and insomnia^32,33^. However, once we controlled for cannabinoid contents, none of the other product characteristics predicted variability in symptom levels. Only THC potency levels showed independent associations with symptom relief and experiences of both positive and negative side effects, with higher levels resulting in larger effects. In contrast, we did not observe an independent link between CBD levels and any of the omnibus symptom effects measured in the current study across nearly 20,000 user sessions.

Variability in cannabinoid profiles may partially explain inconsistent findings in the literature on, for example, the benefits of using cannabis for treating chronic neuropathic pain, with effectiveness observed in some studies^4,34^, but not others^35^. Similarly, while many patient groups (e.g., sleep–disturbed medical cannabis users) have reported a preference for high CBD concentrates^36^, we did not observe any patient outcomes varying by CBD potency levels alone. One possibility is that many of the CBD potency levels displayed on labels of the products consumed in the study were inaccurate (e.g., inflated), as is currently common in the medical cannabis industry^37^. Alternatively, it is possible that CBD has more latent effects than THC (e.g., expanding beyond the 90 minute observation window), has an impact on symptoms infrequently reported in our data, or that CBD’s effects may not lend themselves to perceptual detection and subjective reporting. The phytocannabinoid family of CBDs are known to differ from other cannabinoids such as THC in several ways, including having no affinity to CB1 receptors, serving as an antagonist to GPR55 receptors and as an inverse modulator of the effects of THC and perhaps the endocannabinoid system more generally, as well as functioning as an immuno-suppressant and anti-inflammatory agent^38,39^. Thus, it is possible that while CBD may operate inconspicuously to improve certain health outcomes, the adjunctive consumption of THC is needed to consciously experience or be aware of such effects.

Notwithstanding the innovative nature and potential implications of the study’s findings, our observational, quasi-field experiment had unavoidable limitations, including the lack of a control group, e.g., non-cannabis users with similar symptoms, salient characteristics, past experiences, and voluntary reporting, which could lead to either: a) overestimation of the effectiveness of product characteristics if users who have negative experiences with cannabis are more likely to drop out of the sample by choosing not to use the ReleafApp; or b) underestimation of cannabis’ effectiveness if users fail to use the ReleafApp due to already being satisfied with their product choices and their effects. It is also important to note that the patient-reported outcomes were not cross-referenced with clinical assessments. As with any observational study there is the potential confound of a placebo effect, and given that cannabis products advertised as containing higher THC contents are generally more expensive to consumers, they may be subject to a buyer’s justification effect (magnified appreciation to justify an added expense of purchase). Another limitation is that the ReleafApp may be better suited to tracking the more immediate responses of users of concentrates, flower, and to some extent, tinctures versus the longer to peak effects of edibles and topicals. It is also possible that people who choose not to use the ReleafApp have different experiences with product characteristics than those who do use the app. Another limitation was the inability to distinguish subtleties across product types, such as pipes, which can vary in material construction and potential chemical reactions (e.g., hydrolysis via water pipes). Finally, we anticipate that greater nuances exist in the effects of product characteristics, and particularly, cannabinoid contents across symptom categories, but the current study is limited by our sample size within each respondent subgroup. Future research will capitalize on our ever increasing sample size to analyze the pharmacodynamic interactions of major cannabinoids and other organic compounds including terpenoids, as well as the harm of cannabis production practices. For example, the use of solvents to extract cannabinoids for making concentrates used in making non-flower products (e.g., edibles, tinctures) may place patients at risk for respiratory and cardiovascular problems^40^ and be a cause of increased emergency cases of Cannabinoid Hyperemesis Syndrome^41^.

In conclusion, rapid increases in the popularity of medical cannabis and the associated increase in the number of patients highlight the urgency of investigating and directing effective usage. Cannabis use carries the risk of addiction and short-term impairments in cognitive and behavioral functioning, including the potential for safety issues in the workplace or while driving. However, with preliminary evidence that cannabis may treat an even wider range of conditions than those tracked in this study, including cancer^42,43^, it is imperative that the scientific community develop innovative strategies such as the use of mobile technology for measuring the multidimensional relationships among cannabis product characteristics, patient health conditions, perceived symptom relief, and side effect manifestation.

## Supplementary information

Supplementary Materials

## Data Availability

The data that support the findings of this study are available from MoreBetter Ltd. but restrictions apply to the availability of these data, which were used under license for the current study, and so are not publicly available. Data are however available from the authors upon reasonable request and with permission of MoreBetter Ltd.
