# Supplementary material for: The Association between Cannabis Product Characteristics and Symptom Relief
Source: Sci Rep. 2019 Feb 25;9:2712. doi: 10.1038/s41598-019-39462-1 (PMC6389973; doi:10.1038/s41598-019-39462-1)
Supplement: Supplementary file 1 — Supplementary Materials [file 41598_2019_39462_MOESM1_ESM.docx]

The Association between Cannabis Product Characteristics and Symptom Relief

Sarah S. Stith

Jacob M. Vigil*

Franco Brockelman

Keenan Keeling

Branden Hall

From the University of New Mexico (S.S.S., J.M.V.); the Department of Economics (S.S.S); Department of Psychology (J.M.V); and the MoreBetter Ltd (F.B., K.K.,B.H.).

*Address reprint requests to Dr. Vigil at the University of New Mexico, Department of Psychology, 1 University of New Mexico, MSC03 2220, Albuquerque, NM, 87131-1161, or at [vigilj@unm.edu](mailto:vigilj@unm.edu)

Table S1: Description Statistics for Symptoms

| **Symptom** | **N sessions** | **% of Sample** |
| --- | --- | --- |
| Agitation / Irritability | 1,008 | 5.06 |
| Anxiety | 3,146 | 15.8 |
| Insomnia | 1,371 | 6.89 |
| Muscle Spasms | 302 | 1.52 |
| Stress | 1,908 | 9.58 |
| Depression | 2,029 | 10.19 |
| Pain - Abdominal | 193 | 0.97 |
| Pain - Back | 1,691 | 8.49 |
| Pain - Cramping | 237 | 1.19 |
| Pain - Gastrointestinal | 370 | 1.86 |
| Pain - Headache | 415 | 2.08 |
| Pain – Joint | 832 | 4.18 |
| Pain - Migraine | 415 | 2.08 |
| Pain - Muscle | 848 | 4.26 |
| Pain – Nerve | 567 | 2.85 |
| Pain – Other | 643 | 3.23 |
| Convulsions | 22 | 0.11 |
| Dizziness | 72 | 0.36 |
| Excessive Appetite | 69 | 0.35 |
| Fatigue | 1,155 | 5.8 |
| Impulse | 236 | 1.19 |
| Inflammation | 414 | 2.08 |
| Loss of Appetite | 328 | 1.65 |
| Mood Swings | 208 | 1.04 |
| Nausea | 465 | 2.34 |
| Other | 940 | 4.72 |
| Tremors | 26 | 0.13 |

Table S2: Descriptive Statistics for Side Effects

| **Side Effect** | **Mean** | **Std. Dev.** | **Category** |
| --- | --- | --- | --- |
| Dry Mouth | 0.26 | 0.44 | Negative |
| Foggy | 0.23 | 0.42 | Negative |
| Unmotivated | 0.14 | 0.35 | Negative |
| Scattered | 0.14 | 0.34 | Negative |
| Forgetful | 0.13 | 0.33 | Negative |
| Restless | 0.12 | 0.33 | Negative |
| Red Eyes | 0.11 | 0.31 | Negative |
| Dizzy | 0.11 | 0.31 | Negative |
| Anxious | 0.10 | 0.31 | Negative |
| Irritable | 0.06 | 0.24 | Negative |
| Confused | 0.05 | 0.23 | Negative |
| Headache | 0.05 | 0.22 | Negative |
| Paranoid | 0.03 | 0.18 | Negative |
| Relaxed | 0.63 | 0.48 | Positive |
| Peaceful | 0.54 | 0.50 | Positive |
| Comfy | 0.39 | 0.49 | Positive |
| Chill | 0.38 | 0.49 | Positive |
| Dreamy | 0.34 | 0.47 | Positive |
| Happy | 0.25 | 0.43 | Positive |
| Reflective | 0.25 | 0.43 | Positive |
| Light | 0.24 | 0.43 | Positive |
| Clear | 0.23 | 0.42 | Positive |
| Focused | 0.23 | 0.42 | Positive |
| Tuned | 0.22 | 0.41 | Positive |
| Great | 0.21 | 0.41 | Positive |
| Optimistic | 0.18 | 0.38 | Positive |
| Grateful | 0.15 | 0.36 | Positive |
| Energetic | 0.14 | 0.34 | Positive |
| Creative | 0.13 | 0.34 | Positive |
| Productive | 0.12 | 0.33 | Positive |
| Frisky | 0.09 | 0.29 | Positive |
| Active | 0.07 | 0.26 | Positive |
| High | 0.37 | 0.48 | Context-Specific |
| Thirsty | 0.27 | 0.45 | Context-Specific |
| Sleepy | 0.27 | 0.44 | Context-Specific |
| Tingly | 0.23 | 0.42 | Context-Specific |
| Hungry | 0.23 | 0.42 | Context-Specific |
| Couchlocked | 0.19 | 0.39 | Context-Specific |
| Distracted | 0.15 | 0.35 | Context-Specific |
| Talkative | 0.11 | 0.32 | Context-Specific |
| Silly | 0.09 | 0.29 | Context-Specific |
| Visuals | 0.05 | 0.21 | Context-Specific |

Notes: Context-specific side effects cannot be unambiguously coded as positive or negative.

Table S3: Effects of Product Characteristics on Symptom Relief – Flower Only

|  | **(1)** | **(2)** | **(3)** | **(4)** |
| --- | --- | --- | --- | --- |
|  | **Overall** | **Anxiety** | **Depression** | **Back Pain** |
| THC 10-19% | -0.301** | -0.212 | -1.665*** | 0.196 |
|  | (0.146) | (0.315) | (0.561) | (0.349) |
| THC 19-34% | -0.407*** | -0.466 | -2.726*** | -0.480 |
|  | (0.135) | (0.344) | (0.800) | (0.310) |
| CBD 1-9% | -0.068 | 0.558 | 0.420 | 0.027 |
|  | (0.146) | (0.367) | (0.277) | (0.579) |
| CBD 10-34% | 0.026 | -0.028 | -0.466 | -0.042 |
|  | (0.125) | (0.292) | (0.335) | (0.504) |
| *C. indica* | 0.007 | -0.344* | 0.997** | -0.116 |
|  | (0.103) | (0.186) | (0.477) | (0.271) |
| *C. sativa* | 0.275* | 0.110 | 0.412 | -0.117 |
|  | (0.159) | (0.256) | (0.525) | (0.325) |
| Pipe | -0.014 | 0.222 | -0.647 | -0.097 |
|  | (0.210) | (0.453) | (0.683) | (0.297) |
| Vape | -0.090 | 0.409 | -2.159* | -0.669 |
|  | (0.237) | (0.390) | (1.137) | (0.430) |
| Starting Symptom Level | -0.738*** | -0.609*** | -0.698*** | -0.635*** |
|  | (0.086) | (0.101) | (0.069) | (0.107) |
| Constant | 1.103** | -0.020 | 3.168*** | 0.897 |
|  | (0.544) | (0.621) | (1.106) | (1.189) |
| Observations | 2,763 | 371 | 219 | 283 |
| R-squared | 0.366 | 0.357 | 0.510 | 0.387 |
| N Users | 652 | 211 | 136 | 130 |

Notes: Only sessions using flower are included. Regressions are run overall and by symptom category. Each column represents a separate regression. Regressions control for individual user fixed effects. The Concentrate is relative to Flower, *C. indica* and *C. sativa* are relative to Hybrid, THC categories are relative to THC 0-9%, CBD categories are relative to CBD 0%, and Pipe and Vape are relative to Joint. Standard errors are clustered at the user level (shown in parentheses). *** p<0.01, ** p<0.05, * p<0.1

Table S4: Robustness Checks – Subsample Analyses to Avoid High Frequency Users Driving the Results

|  | (1) | (2) |
| --- | --- | --- |
|  | **Total Sessions < 10** | **Within 1st 5 Sessions** |
| Concentrate | 1.474 | 1.358 |
|  | (1.041) | (0.883) |
| Tincture |  |  |
|  |  |  |
| *C. indica* | -0.495* | -0.566** |
|  | (0.253) | (0.228) |
| *C. sativa* | 0.120 | 0.099 |
|  | (0.339) | (0.257) |
| THC 10-19% | -0.356 | -0.515 |
|  | (0.437) | (0.363) |
| THC 19-34% | -0.470 | -0.350 |
|  | (0.352) | (0.340) |
| THC 35%+ | -2.526*** | -1.755** |
|  | (0.884) | (0.701) |
| CBD 1-9% | -0.007 | 0.207 |
|  | (0.316) | (0.376) |
| CBD 10-34% | -0.210 | 0.282 |
|  | (0.285) | (0.371) |
| CBD 35%+ | 0.898** | 1.064** |
|  | (0.394) | (0.509) |
| Starting Symptom Level | -0.722*** | -0.727*** |
|  | (0.047) | (0.043) |
| Constant | 1.264*** | 0.891* |
|  | (0.467) | (0.470) |
| Observations | 1,133 | 1,415 |
| R-squared | 0.406 | 0.439 |
| N Users | 612 | 738 |

Notes: Regressions control for individual user fixed effects. Column (1) includes only users who had completed fewer than ten sessions by the time of our analysis. Column (2) includes only the first five sessions for all users. Concentrate and Tincture are relative to Flower, *C. indica* and *C. sativa* are relative to Hybrid, THC categories are relative to THC between 0 and 9%, and CBD categories are relative to CBD =0%. Standard errors are clustered at the user level (shown in parentheses). *** p<0.01, ** p<0.05, * p<0.1
